# Methadone, Buprenorphine, and Clonidine Attenuate Mitragynine Withdrawal in Rats

**DOI:** 10.3389/fphar.2021.708019

**Published:** 2021-07-12

**Authors:** Rahimah Hassan, Sasidharan Sreenivasan, Christian P. Müller, Zurina Hassan

**Affiliations:** ^1^ Centre for Drug Research, Universiti Sains Malaysia, Minden, Malaysia; ^2^ Institute for Research in Molecular Medicine, Universiti Sains Malaysia, Minden, Malaysia; ^3^ Section of Addiction Medicine, Department of Psychiatry and Psychotherapy, University Clinic, Friedrich-Alexander-University Erlangen-Nuremberg, Erlangen, Germany; ^4^ Addiction Behaviour and Neuroplasticity Laboratory, National Neuroscience Institute, Singapore, Singapore

**Keywords:** mitragynine, kratom, withdrawal, replacement, methadone, burprenorphine, clonidine

## Abstract

**Background:** Kratom or *Mitragyna speciosa* Korth has been widely used to relieve the severity of opioid withdrawal in natural settings. However, several studies have reported that kratom may by itself cause dependence following chronic consumption. Yet, there is currently no formal treatment for kratom dependence. Mitragynine, is the major psychoactive alkaloid in kratom. Chronic mitragynine treatment can cause addiction-like symptoms in rodent models including withdrawal behaviour. In this study we assessed whether the prescription drugs, methadone, buprenorphine and clonidine, could mitigate mitragynine withdrawal effects. In order to assess treatment safety, we also evaluated hematological, biochemical and histopathological treatment effects.

**Methods:** We induced mitragynine withdrawal behaviour in a chronic treatment paradigm in rats. Methadone (1.0 mg/kg), buprenorphine (0.8 mg/kg) and clonidine (0.1 mg/kg) were i.p. administered over four days during mitragynine withdrawal. These treatments were stopped and withdrawal sign assessment continued. Thereafter, toxicological profiles of the treatments were evaluated in the blood and in organs.

**Results:** Chronic mitragynine treatment caused significant withdrawal behaviour lasting at least 5 days. Methadone, buprenorphine, as well as clonidine treatments significantly attenuated these withdrawal signs. No major effects on blood or organ toxicity were observed.

**Conclusion:** These data suggest that the already available prescription medications methadone, buprenorphine, and clonidine are capable to alleviate mitragynine withdrawal signs rats. This may suggest them as treatment options also for problematic mitragynine/kratom use in humans.

## Introduction

*Mitragyna speciosa* Korth or kratom is traditionally used in South-East Asia, particularly in Thailand and Malaysia, for its psychoactive effects. Kratom leaves have been claimed to have both psychostimulant- and opium-like narcotic effects. At low dose it acts as a stimulant, while being sedative at high doses (Jansen and Prast, 1988). Locals historically use kratom to combat exhaustion and survive working under bright sunlight through its psychostimulant-like effect.

Furthermore, kratom is also used to self-medicate for opioid withdrawal symptoms and as a replacement for heroin and morphine (Beckett et al., 1965; Grundmann, 2017). Currently, kratom emerged in the self-management of pain and opioid withdrawal, especially in the United States (Prozialeck, 2016; Grundmann, 2017). Kratom can be easily purchased on the internet. It has cheap prices and being marketed in many forms, from tablet to extract, in leaf form, as topical creams, balms or tinctures (Stanciu et al., 2019). In the United States, kratom is marketed and regulated as a dietary or herbal supplement. Individuals apply it for management of anxiety, pain, opioid use disorder, and depression (Boyer et al., 2008; Grundmann, 2017; Coe et al., 2019). Nevertheless, complications have arisen from this. The poorly regulated botanical and dietary supplement market which is also made up of adulterated products and where kratom products are sold, may partially account for the fatal cases (Obeng et al., 2020) that arise from their consumption. Indeed, death from kratom ingestion is exceedingly uncommon (Eastlack et al., 2020). Nonetheless, it can occur as a result of poly-substance abuse, which can contribute to an increased mortality risk (Neerman et al., 2013). Additionally, the Center for Disease Control and Prevention (CDC) reported 152 kratom-related deaths between the period of July 2016 and December 2017, all of which contained polydrug (Kuehn, 2019). Furthermore, 156 deaths have been linked to kratom use, with 87% being linked to polydrug use (Corkery et al., 2019).

Whilst kratom has benefits, it also has been reported that they can cause dependence and addiction-like symptoms after long-term consumption in humans (Vicknasingam et al., 2010; Ahmad and Aziz, 2012; Singh et al., 2014; Singh et al., 2018a; Müller et al., 2020; Anand and Hosanagar, 2021). Kratom withdrawal symptoms include, jerky movement, muscle ache, aggression, wet nose, and hostility in natural settings (Suwanlert, 1975). Furthermore, kratom users have been reported to have difficulties in combating kratom withdrawal while trying to stop its consumption (Ahmad and Aziz, 2012; Singh et al., 2014).

Kratom leaves contain over 40 alkaloids where mitragynine is the main indole alkaloid (Adkins et al., 2011; Yearsley, 2016). For this reason, we believed that mitragynine might be one of the alkaloids that modulate the effects in kratom. Currently, there is no specific treatment implemented in managing kratom dependence and withdrawal symptoms. Since no standardized treatment for kratom dependence is currently applied, therefore, the present study aims to investigate whether the available prescription drugs for opioid management; methadone, buprenorphine and clonidine, would mitigate the withdrawal symptoms caused by chronic mitragynine exposure.

## Materials and Methods

### Animals

All animal experiments were performed in accordance with approved guidelines and regulations of the Universiti Sains Malaysia (USM) Institutional Animal Care and Use Committee (USM IACUC) [Reference number: USM/Animal Ethics Approval/2016/ (Santiago and Edelman, 1985) (736)]. Animals were purchased from Animal Research and Service Centre (ARASC), Universiti Sains Malaysia, Penang, Malaysia. All 30 tested animals were male Sprague-Dawley rats (200–300 g). They were naive and used in a single experiment only. Animals were socially housed in groups of six per cage under standard laboratory conditions, with temperature-controlled environment (24 ± 1°C) during habituation and were then placed individually prior to the treatment. The room was maintained on a 12 h light/12 h dark normal cycle (lights on from 07:00 to 19:00 h). Animals were handled for one week prior to commencement of the experiments. Food and water were available *ad libitum*.

### Drugs Preparation

Methadone hydrochloride, buprenorphine hydrochloride and clonidine hydrochloride were purchased from Sigma Chemicals Co. (United States). Mitragynine was extracted, isolated and verified from fresh leaves of *Mitragyna speciosa* at the Centre for Drug Research, Universiti Sains Malaysia as described previously (Utar et al., 2011). Purified mitragynine was confirmed by high performance liquid chromatography (HPLC) and proton nuclear magnetic resonance (^1^H-NMR) (400 MHz) analysis (Jamil et al., 2013). Mitragynine obtained by this procedure was approximately 98% pure (Hassan et al., 2019). Fresh stocks of methadone, buprenorphine, mitragynine and clonidine were prepared daily according to the weight of animals in the experimental design. They were dissolved in vehicle (20% (v/v) Tween 80 which was diluted with physiological saline (0.9% NaCl); Sigma Aldrich, United Kingdom) and injected intraperitoneally (i.p.).

### Experimental Design for Replacement Treatment in Mitragynine Withdrawal Model

The previously established mitragynine withdrawal model was used (Hassan et al., 2021). Mitragynine (30 mg/kg, i.p.) was injected once per day over a period of 14 days. The vehicle group received 20% Tween 80 also once daily for 14 days. For both the vehicle- and mitragynine groups, withdrawal symptoms were assessed on day 15, twenty-four hours after abstinence from the drug. In this model, the effectiveness of the available prescription medications, methadone, buprenorphine and clonidine, were accessed. All the replacement treatments were applied for four days and then abruptly stopped on day 5 in order to determine whether or not the mitragynine withdrawal signs will resurface. This design followed the replacement routine described by Hassan et al. (2020). In a subsequent test, we examined hematological, biochemical and histopathological effects of the mitragynine and the replacement treatments.

### Experiment I: Methadone, Buprenorphine and Clonidine Replacement Treatments in Mitragynine Withdrawn Rats.

The methadone dose used in the present study (1.0 mg/kg, i. p.) was selected to be in the pharmacological range and below the LD_50_ value to reduce fatality risk (McCormick et al., 1984; Chevillard et al., 2010). Methadone was dissolved in vehicle (20% Tween 80; Sigma Aldrich, United Kingdom) and injected i. p. 10 min before withdrawal testing, i.e., 23 h 50 min after the last mitragynine dose (Chevillard et al., 2010). Thereafter, methadone was repeatedly administered every 8 h (Chevillard et al., 2010) for four days of replacement treatment.

The buprenorphine dose used in the present study (0.8 mg/kg, i.p.) was selected to be in the pharmacological range of previous studies (Cowan et al., 1977; McLaughlin and Dewey, 1994; Morgan et al., 1999). Buprenorphine was dissolved in vehicle (20% Tween 80; Sigma Aldrich, United Kingdom) and injected i.p. 30 min before withdrawal testing, i.e., 23.5 h after the last mitragynine dose (Cowan et al., 1977). Buprenorphine was then administered every 12 h (Schaap et al., 2012) for four days.

The clonidine dose used in the present study (0.1 mg/kg, i.p.) was based on the previous study by Tierney et al. (1988). In the present study, clonidine was dissolved in vehicle (20% Tween 80; Sigma Aldrich, United Kingdom) and injected i.p. 10 min before withdrawal testing, i.e., 23 h 50 min after the last mitragynine dose (Tierney et al., 1988; Sireesha et al., 2015). Thereafter, clonidine was administered every 12 h for four days (Feily and Abbasi, 2009; Moayeri et al., 2018).

### Assessment of Withdrawal Behaviour

Trained observers who were blind to treatment and time points, scored all behaviours from video and counted the frequency of the signs of spontaneous withdrawal: chewing, head shakes, exploring, digging, yawning, teeth chattering, wet dog shakes, and writhing as well as checked the signs of squeaking on touch, hostility on handling, and diarrhoea. The observers showed an inter-rater reliability for this scoring of r = 0.99. The recording was started once the animals were placed in an open field test box for 30 min and the withdrawal behaviour were scored. The test was performed daily for 4 days of replacement therapy and on day 5 when the treatment had stopped. The withdrawal behaviours were distinguished as “counted signs”, including chewing, head shakes, exploring, digging, yawning, teeth chattering, wet dog shakes, writhing, and as “checked signs”, including squeaking on touch, hostility on handling and diarrhoea. Thereby, counted signs and checked signs were further processed by multiplying with the respective weighting factors for evaluation of the severity of withdrawal signs using the previously described scoring method by Hassan et al. (2020), Rahman et al. (2002), and Sabetghadam et al. (2013) (Table 1).

**TABLE 1:** The counted signs and checked signs with the respective weighing factors for the evaluation of mitragynine-withdrawal severity in rats.

Counted signs	Weighing factors	Checked signs (Checked every 10 min)	Weighing factors
Chewing	2	Squeaking on touch	1
Head shakes	2	Hostility on handling	1
Exploring	1	Diarrhoea	1
Digging	2	—	—
Yawning	2	—	—
Teeth chattering	2	—	—
Wet dog shakes	2	—	—
Writhing	2	—	—

### Experiment II

#### Hematological Analysis

After behavioural studies ended on day 5, all the treated rats were euthanized with sodium pentobarbital (100 mg/kg) intraperitoneally. Blood samples were collected via cardiac puncture and transferred into tubes containing ethylenediamine tetraacetic acid (EDTA). Blood samples were analysed for several hematological parameters such as red blood cell count (Total RBC), haemoglobin, percentage of packed cell volume (PCV%), mean corpuscular volume (MCV), mean corpuscular haemoglobin (MCH), mean corpuscular haemoglobin concentration (MCHC), percentage of red cell distribution width (RDW%), total of white blood cell count (WBC), percentage of lymphocyte, monocytes, eosinophils, basophils, and platelet counts (PLT).

#### Biochemical Analysis

The collected blood was transferred into serum-separating tubes for biochemical analysis. The biochemical parameters analysed were total bilirubin, aspartate amino transferase (AST), alanine aminotransferase (ALT), alkaline phosphatase, sodium, potassium, chloride, urea, creatinine, total cholesterol, triglycerides, calcium, phosphorus, total protein, albumin, globulin and albumin/globulin ratio (A/G ratio).

#### Histopathological Analysis

On day 5, animal tissue samples of targeted organs (heart, lung, kidney, liver) were harvested after behavioural testing. The tissues were stained using Hematoxylin and Eosin. Then, the slides were viewed under light microscope equipped with a digital camera. The sections were analysed for structural changes, degenerative alterations, necrosis and signs of inflammation.

### Statistical Analysis

All data were expressed as mean ± standard error of the mean (SEM). The substitution treatments were analysed by two-way ANOVA for repeated measures with test “day” as within factor and treatment combination as a “treatment” between factors. In order to analyse single group differences on each treatment day, pre-planned comparisons were calculated using Bonferroni test (Ramsey and Edwards, 1993). Hematological and biochemical parameters were analysed by one-way repeated measures ANOVA and Bonferroni post-hoc tests. Each of the hematological and biochemical parameters as fixed factor or within factor, whereas treatment combination is between factors. A significance level of *p* < 0.05 was used to test for statistical significance. GraphPad Prism 8.0 software (GraphPad Software Inc., La Jolla, CA, United States) was used to perform the statistics.

## Result

### Experiment I: Methadone, Buprenorphine and Clonidine Replacement Treatments in Attenuating Withdrawal Effects Due to Mitragynine

**FIGURE 1:**
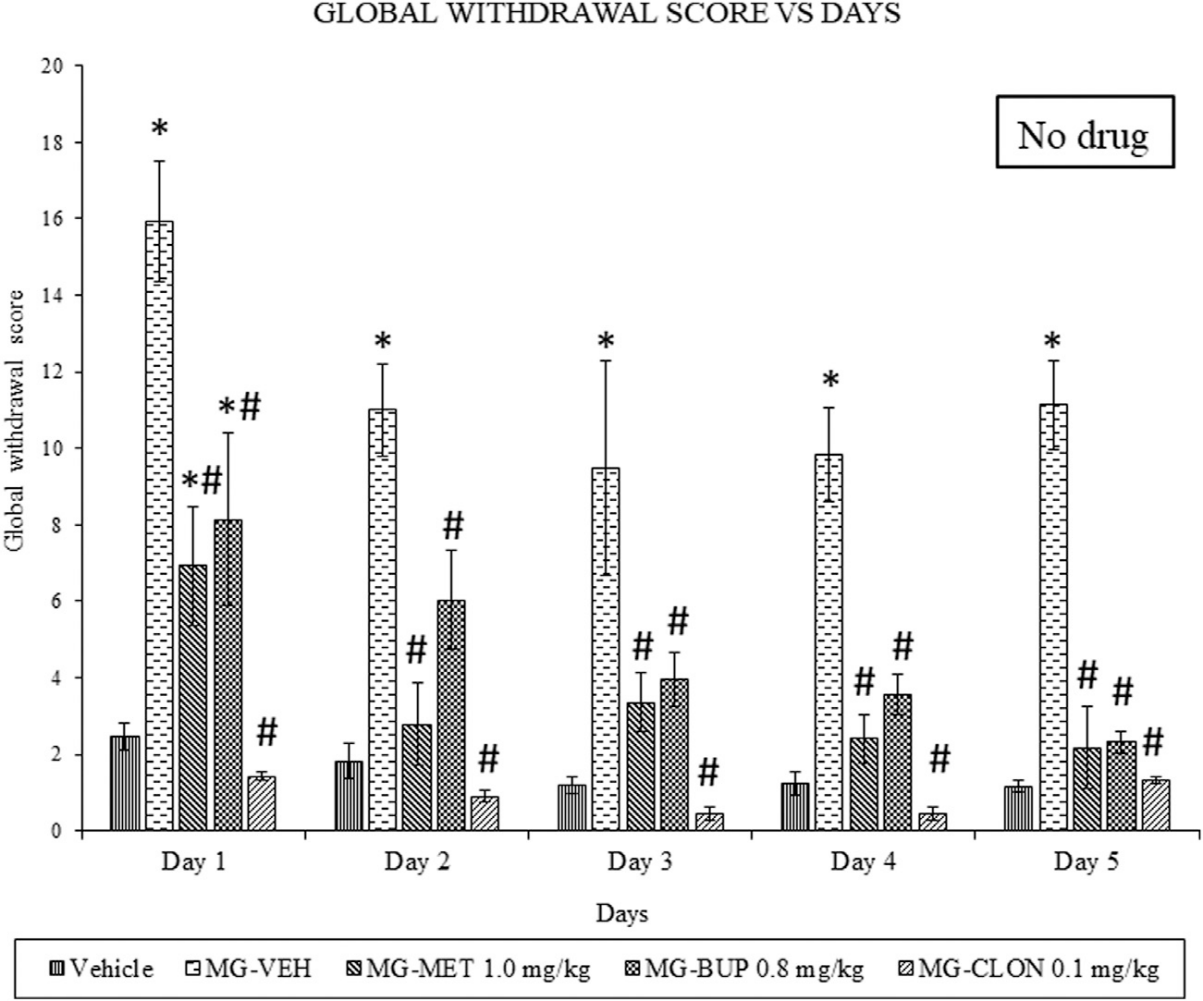
Methadone (MET), Buprenorphine (BUP) and Clonidine (CLON) reduced behavioural signs of mitragynine withdrawal in rats. Data represent means (±SEM) of global withdrawal signs (*n* = 6/group; *p < 0.05, vs. Vehicle, #*p* < 0.05 vs. mitragynine-vehicle, MG-VEH).

We found that chronic mitragynine treatment induced significant withdrawal behaviour on all 5 days after cessation of administration (Figure 1). Methadone, buprenorphine, as well as clonidine significantly attenuated the mitragynine withdrawal effects on all 4 days of the replacement treatment, and on day 5 when no treatment was given. A two-way ANOVA showed significant treatment (F_4,75_ = 77.69, *p* < 0.0001) and day effects (F_4,75_ = 9.327, *p* < 0.0001), but no significant treatment × day interaction (F_16,75_ = 1.222, *p* = 0.2721). On day 1, methadone significantly reduced withdrawal signs as compared to vehicle and mitragynine groups (*p* < 0.05) (Figure 1). From day 2 to day 4 as well as on day 5 on which no replacement treatment was given, there was a significant difference compared to the mitragynine group (*p* < 0.05), but not to the vehicle group (*p* > 0.05). Buprenorphine also significantly alleviated the withdrawal signs as compared to vehicle and mitragynine groups on day 1 (*p* < 0.05) (Figure 1). A significant mitigation of withdrawal signs was also observed from day 2 to day 5, as compared to the mitragynine group (*p* < 0.05), but not compared to the vehicle group (*p* > 0.05). The strongest suppression of mitragynine withdrawal effects was observed after clonidine treatment (day 1–5; vs. mitragynine-vehicle: *p* < 0.05). From day 1 to day 5, no significant difference was shown between the mitragynine-clonidine treated group compared to the vehicle group (*p* > 0.05) and significant result has been revealed as compared to mitragynine group (*p* < 0.05).

### Experiment II

#### Hematological and Biochemical Analysis

Hematological and biochemical analyses of the blood samples were taken on day 5 and results are displayed in Tables 2, 3, respectively. The references range value of both hematological and biochemical analyses were based on these following studies Nugraheni et al. (2017), He et al. (2017), Houtmeyers et al. (2016) and Petterino and Argentino-Storino (2006). The hematological analysis revealed a significant increase in mean corpuscular hemoglobin concentration (MCHC). This was higher than the reference range in the mitragynine-methadone group and significantly increased compared to the vehicle control group (*p* < 0.05). A significant increase in platelet count was found in the mitragynine-buprenorphine group (*p* < 0.05 vs vehicle; Table 2). The biochemical analysis revealed a significant reduction of total cholesterol in the mitragynine-methadone and mitragynine-buprenorphine groups (*p* < 0.05) compared to vehicle control (Table 3).

**TABLE 2:** Hematological analysis on day 5 in mitragynine replacement treatments.

Replacement groups	Vehicle	Mitragynine – Vehicle	Mitragynine 1 mg/kg Methadone	Mitragynine 0.8 mg/kg Buprenorphine	Mitragynine 1.1 mg/kg Clonidine	References range value
Total RBC (x 10^∧^12/L)	6.97 ± 0.24	8.23 ± 0.6	7.93 ± 0.35	8 ± 0.17	7.93 ± 0.37	6.39–8.01
Hemoglobin (gm/L)	155.67 ± 8.19	168 ± 12.7	168 ± 4.36	159 ± 8.02	156.67 ± 9.61	135–159
PCV (%)	49.33 ± 1.86	47 ± 0.04	45 ± 1.73	46.33 ± 1.76	43.67 ± 0.02	42–49
MCV (fL)	58.33 ± 2.03	57 ± 1.73	58 ± 0.58	59 ± 2.08	55 ± 1.15	58.01–67.00
MCH (pg)	20.67 ± 0.33	20.33 ± 0.33	21 ± 0.58	19 ± 1.53	19.67 ± 0.33	18.70–21.20
MCHC (g/L)	316.67 ± 8.82	360 ± 5.77	376.67 ± 8.82*	320 ± 20	360 ± 5.77	310–336
RDW (%)	15.17 ± 0.42	14.93 ± 0.94	14.93 ± 0.12	15.4 ± 0.58	14.93 ± 0.26	13.03–16.57
Total WBC (x 10^9/L)	4.2 ± 1.18	4.8 ± 1.29	4.4 ± 0.71	6.47 ± 0.18	5.13 ± 0.96	3.00–9.22
Lymphocytes (%)	76.33 ± 2.03	66 ± 5.86	65.67 ± 6.64	76.33 ± 1.45	69 ± 1.53	51.8–89.7
Monocytes (%)	2.67 ± 0.67	0.33 ± 0.33	3 ± 0.58	2.67 ± 0.67	0.67 ± 0.33	1.3–6.0
Eosinophils (%)	0 ± 0	0.33 ± 0.33	0.33 ± 0.33	0.58 ± 0.01	0 ± 0	0.5–7.2
Basophils (%)	0 ± 0	0 ± 0	0 ± 0	0 ± 0	0 ± 0	0–0.6
Platelet count (x 10^∧^9/L)	682 ± 112.65	529 ± 62.69	702.33 ± 18.89	946.33 ± 3.53#	483.33 ± 39.54	529.0–1,383.0

Total RBC, red blood cell count; PCV%, percentage of packed cell volume; MCV, mean corpuscular volume; MCH, mean corpuscular haemoglobin; MCHC, mean corpuscular haemoglobin concentration; RDW%, percentage of red cell distribution width; WBC, total of white blood cell count, Lymphocytes (%), Percentage of lymphocyte; Monocytes (%), Percentage of monocytes; Eosinophils (%), Percentage of eosinophils; Basophils (%), Percentage of basophils; PLT, Platelet counts.

Data represent means ± SEM of n = 6 rats/group *p < 0.05 vs. Vehicle, #*p* < 0.05 vs. Mitragynine-Vehicle, analysed by one-way repeated measures ANOVA and Bonferroni post-hoc test.

**TABLE 3:** Biological analysis on day 5 in mitragynine replacement treatments.

Replacement groups	Vehicle	Mitragynine – Vehicle	Mitragynine 1 mg/kg Methadone	Mitragynine 0.8 mg/kg Buprenorphine	Mitragynine 0.1 mg/kg Clonidine	References range value
Total bilirubin (umol/L)	1.71 ± 0	2.28 ± 0.57	1.71 ± 0	2.28 ± 0.57	2.28 ± 0.57	0.0–5.1
Aspartate aminotransferase, AST (U/L)	117 ± 4.58	205.33 ± 36.08	169 ± 20.03	147 ± 1	220.33 ± 35.57	56.1–201.8
Alanine aminotransferase, ALT (U/L)	59 ± 6.11	74 ± 2.08	53 ± 2.31	58.33 ± 3.38	55.33 ± 6.69	34.9–218.1
Alkaline phosphatase (U/L)	299.67 ± 28.01	381.67 ± 119.7	337.33 ± 48.55	421 ± 86.97	253.33 ± 32.69	131.6–459.0
Sodium (mmol/L)	141.67 ± 1.45	139.33 ± 0.67	141 ± 1.73	143.67 ± 0.67	140.33 ± 0.88	121.9–162.6
Potassium (mmol/L)	6.7 ± 1.26	7.67 ± 0.12	7.8 ± 0.85	6.77 ± 0.24	7.1 ± 0.15	4.0–8.0
Chloride (mmol/L)	102.33 ± 0.88	102.33 ± 0.33	103 ± 0.58	104 ± 0	104.67 ± 1.2	81.5–104.0
Urea (mmol/L)	9.80 ± 0.3	8.33 ± 0.45	8.05 ± 0.11	14.45 ± 6.04	10.03 ± 0.45	4.32–34.4
Creatinine (umol/L)	49.87 ± 2.35	46.93 ± 0.59	46.35 ± 1.63	73.92 ± 33.93	43.71 ± 1.28	35.4–79.6
Total cholesterol (mmol/L)	1.70 ± 0.08	2.1 ± 0.11	1.59 ± 0.09#	1.47 ± 0.07#	1.74 ± 0.11	0.68–1.77
Triglycerides (mmol/L)	0.93 ± 0.19	0.82 ± 0.19	0.98 ± 0.09	0.99 ± 0.14	0.65 ± 0.15	0.23–0.99
Calcium (mmol/L)	2.41 ± 0.11	2.33 ± 0.11	2.48 ± 0.01	2.38 ± 0.03	2.28 ± 0.06	2.1–2.9
Phosphorus (mmol/L)	2 ± 0.30	2.36 ± 0.28	2.13 ± 0.25	2.22 ± 0.09	2.35 ± 0.17	1–3.94
Total protein (g/L)	70 ± 4.36	73.67 ± 1.76	65.67 ± 0.88	67.67 ± 1.86	69.67 ± 3.38	52–71
Albumin (g/L)	27 ± 2.08	28 ± 0.58	25.67 ± 0.88	26 ± 1.53	26.33 ± 1.33	26.85–34.55
Globulin (g/L)	43.33 ± 2.33	45.67 ± 2.19	40 ± 1.53	41.67 ± 1.2	43.33 ± 3.18	13–48
Albumin/Globulin ratio (g/L)	0.6 ± 0.06	0.63 ± 0.07	0.63 ± 0.03	0.63 ± 0.03	0.63 ± 0.07	0.6–1.21

Data represent means ± SEM of n = 6 rats/group *p < 0.05 vs. Vehicle, #*p* < 0.05 vs. Mitragynine-Vehicle, analysed by one-way repeated measures ANOVA and Bonferroni post-hoc test.

#### Histopathology Analysis

The results of histopathological examination of the transverse sections of heart, lung, kidney, and liver are shown in Table 4. In general, there were no differences between the vehicle and the treatment groups by cross-examination of the microscopic structures of the heart, kidney and liver. However, the lung structure revealed a slight difference effect in the alveoli size in all replacement treated groups when compared to the vehicle group (Table 4).

**TABLE 4:** The microscopic structures of the organs in mitragynine replacement treatments. 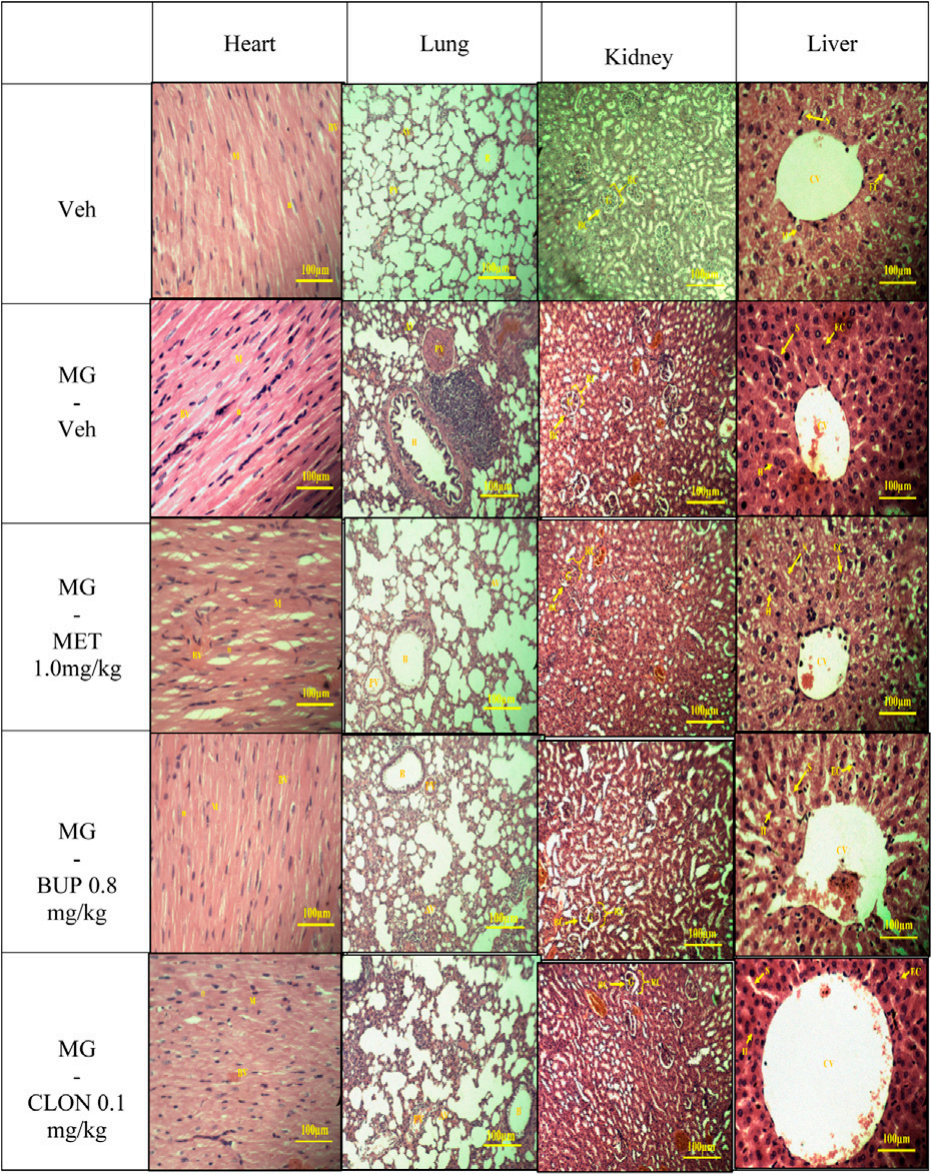

## Discussion

When a person is compelled to take a drug on a regular or continuous basis in order to achieve psychic effects on mental state, such as euphoria, and to avoid physical discomfort (withdrawal), this is referred to as drug dependence (Gupta and Gupta, 2018; Müller, 2020). Dependence in kratom is well documented in human (Suwanlert, 1975; Vicknasingam et al., 2010; Ahmad and Aziz, 2012; Singh et al., 2014). Nevertheless, although kratom has been reported to cause dependence and withdrawal signs, these symptoms are usually milder than after opiate withdrawal (Prozialeck, 2016; Saingam et al., 2016; Grundmann, 2017; Singh et al., 2018b; Swogger and Walsh, 2018). Singh et al. (2018b) reported that kratom depended patients did not seek for medication as kratom withdrawal symptoms were mostly rather mild and only lasted between one to three days. A widely held view is that kratom is not as risky as opioid drugs and that the danger can be outweighed by the potential benefits (Prozialeck, 2016). However, aggressive kratom consumption pattern may cause escalation of consumption and more severe withdrawal signs (Müller et al., 2021). Indeed, withdrawal periods are highly aversive, which makes it hard for an individual to maintain abstinence (Swogger and Walsh, 2018).

At the moment, there is no particular treatment for kratom dependence and withdrawal symptoms. Nonetheless, several studies have been conducted in which buprenorphine was substituted for kratom (Buresh, 2018; Bowe and Kerr, 2020; Lei et al., 2021). The recent study by Weiss and Douglas (Weiss and Douglas, 2021) demonstrated that patients who used less than 20 g of kratom per day could begin opioid agonist therapy with 4/1 mg-8/2 mg buprenorphine-naloxone/day, whereas patients who used more than 40 g of kratom per day could begin with 12/3 mg-16/4 mg buprenorphine-naloxone/day. Kratom leaves do contain more than 40 alkaloids, with mitragynine being the most common indole alkaloid (Adkins et al., 2011; Yearsley, 2016). As a result, we presumed that mitragynine was one of the alkaloids that modulated the effects of kratom. However, one feature that should be noted is that the alkaloid contents vary according to geographical regions and seasons (Hassan et al., 2013). Stages in leaf maturity too are a contributing factor (Raffa, 2015). Studies have shown that mitragynine content is much more abundant in Thai kratom as compared to the Malaysian species, amounting to 66.2 and 12%, respectively (Takayama et al., 1998; Takayama, 2004). This would suggest that kratom substitution treatment is difficult to be evaluated due to differences in mitragynine content. Therefore, the current study will focus on mitragynine rather than kratom dependence. Mitragynine is a psychoactive substance which also can produce dependence for which no pharmacotreatment is available yet. Mitragynine has been reported to cause physical dependence after spontaneously mitragynine withdrawal (Hassan et al., 2021) and after naloxone-precipitated withdrawal (Harun et al., 2020) in animal studies. In addition, Yusoff et al. (2016) also reported that chronic mitragynine withdrawal triggers anxiety-like behaviour in rats. Because there is presently no established treatment for kratom dependence, the present study investigated the effectiveness of methadone, buprenorphine, and clonidine, in attenuating the withdrawal symptoms induced by persistent mitragynine exposure. Buprenorphine, methadone, and clonidine have been identified as the most effective opioid detoxification medications (Meader, 2010). Thus, that is why we used those treatments in the current study. In addition, the opioid and non-opioid mitragynine receptors that may be involved in mitragynine withdrawal were also being considered, and the drug used in the current study was influenced by them. This research is, to the best of our knowledge, the first study on the effects of methadone, buprenorphine and clonidine on mitragynine withdrawal symptoms.

We recently published a rat mitragynine withdrawal model (Hassan et al., 2021). Thus therefore, we used this model in the following experiments in this present work. Overall, this study showed a marked reduction in withdrawal symptoms in mitragynine withdrawn rats receiving methadone, buprenorphine and clonidine. In addition, no resurface of the withdrawal symptoms was seen on day 5 of the cessation (Figure 1). This suggests that methadone, buprenorphine and clonidine are capable in alleviating mitragynine withdrawal signs during and after the replacement therapy. This may also imply that mitragynine withdrawal modulates the same receptors as the mechanisms of action in methadone, buprenorphine and clonidine.

Mitragynine has been proved to bind at opioid receptor, which has been demonstrated by several researchers via *in vivo and in vitro* studies*.* Matsumoto et al. (Matsumoto et al. (1996a) has reported that involvement of opioid receptors in the action of mitragynine. Mitragynine acting on opioid systems can also be observed in the studies of ileum motility inhibition (Watanabe et al., 1997) and inhibition of gastric acid secretion (Tsuchiya et al., 2002) that has been reversed by naloxone. Furthermore, Shamima et al. (2012) also reported the antinociceptive effects of mitragynine when it showed a significant decrease in the latency time compared to mitragynine alone after intraperitoneally administered of naloxone (non-selective opioid antagonist) and naltrindole (delta-opioid antagonist). Naloxonazine, a µ1-receptor antagonist did reduce the antinociceptive effect of mitragynine, but it is not statistically significant, which indicates that mitragynine may not only act specifically on µ1-receptor. However, norbinaltorpimine (norBNI) (i.p.) partially blocked the effect of mitragynine and significantly decreased the latency time when compared with mitragynine alone from 30 to 60 min, but not up to 120 min time, indicate that mitragynine may partially act via kappa opioid receptor (Shamima et al., 2012). In addition, Kruegel et al. (2016) has demonstrated that mitragynine acts as a partial agonist at the human mu-opioid receptor (MOP) and competitive antagonists at kappa-(KOP) and delta- (DOP) opioid receptors in *in intro* study. Indeed, this matter is still in question with the facts about whether mitragynine fully or partially works on the mu-opioid receptor remaining uncertain. Nonetheless, Yusoff et al. (2017) revealed that mitragynine-induced CPP establishment, but not expression, is mediated by an opioid receptor mechanism. Moreover, recently, the effect of naloxone on precipitated of mitragynine withdrawal effects was described by Harun et al. (2020), suggesting that the mu-opioid receptor is responsible for the development of mitragynine dependence.

However, Hiranita et al. (2019) suggested that mitragynine is not mediated through opioid receptor, as naltrexone did not antagonize the effects of mitragynine. It was proposed that activation of serotonergic and noradrenergic pathways along the spinal contributed to the antinociceptive activity of mitragynine (Matsumoto et al., 1996b). It also reported to bind at non-opioid receptors includes alpha-_2_ adrenergic receptors, adenosine A_2_a receptors, dopamine D_2_ receptors, and the serotonin receptors 5-HT_2C_ and 5-HT_7_ (Boyer et al., 2008; Prozialeck et al., 2012; Kruegel and Grundmann, 2018). Currently, an *in vitro* study evaluated the adrenergic effects by mitragynine using human monoclonal receptors expressed in Chinese hamster ovary (CHO) cells and revealed that mitragynine is a partial agonist at alpha _1A_ and _D_ adrenergic receptors (Obeng et al., 2020).

Methadone and buprenorphine are both opioids, acting fully and partially on mu-opioid receptor, respectively (Kleber, 2007). Methadone and buprenorphine have been approved by the FDA in opioid replacement therapy (Bart, 2012). Methadone safety is well documented and proven (Kreek, 1973). However, if taken beyond the tolerance of the person, methadone could cause respiratory depression (Bart, 2012). The respiratory depression could also be fatal in the event of overdose since there is no ceiling level to it (Mattick et al., 2008). Moreover, unknown drug-drug interaction can also lead to death. Records indicate that methadone patients who use other controlled drugs in conjunction with methadone commonly face serious adverse effects (Mattick et al., 2008). Since the 1970s, buprenorphine has been accepted as an alternative treatment of opioid dependence (Mattick et al., 2008). Buprenorphine is also used as an analgesic in acute pain management. Buprenorphine has ceiling effects on respiratory depression (Ling and Compton, 2005). In contrast, a full agonist for respiratory depression which caused a robust decrease in respiratory ventilation following intracerebroventricular buprenorphine administration has been reported by Kuo et al. (2015). Moreover, buprenorphine also does not appear to exhibit a ceiling effect for analgesia (Dahan et al., 2006). At high dose, it antagonizes the analgesic effects of other opioids thereby complicating management of pain in patients maintained on high-dose buprenorphine (Heinzerling et al., 2019). Its antagonist properties can also cause a precipitation of acute opiate withdrawal effects if administered to an individual who is physically dependent on opioids.

Clonidine is a non-opioid drug, that is a partial agonist of alpha_2_-adrenergic receptors (Giovannitti et al., 2015; Arora and Vohora, 2016). It was used in opioid substitution treatment over the years (Jamadarkhana and Gopal, 2010). It has analgesic effects (Kariya et al., 1998) and has been reported to decrease opioid dosage without affecting the quality of analgesia (Rostaing et al., 1991). It can reduce opiate withdrawal signs in inpatient and outpatient settings (Washton and Resnick, 1981; Tierney et al., 1988), via binding to α_2_-autoreceptors in the locus coeruleus and suppressing hyperactivity during withdrawal (Kleber, 2007). However, clonidine has adverse effects, particularly hypotension, which can restrict optimal clonidine dose for opioid withdrawal. It was reported to cause rebound hypertensive episodes in long-term clonidine therapy but proved as safe in short-term use (Kariya et al., 1998). Kleber (2007) also showed that clonidine can be used to treat residual mild withdrawal symptoms for a few days to a week as long as the patient does not become hypotensive. Nevertheless, clonidine has been reported to be less effective compared to methadone during early opioid detoxification phase when withdrawal symptoms were more pronounced and patients more likely to drop out (Mattick and Hall, 1996). On the other hand, clonidine showed in clinical trial a similar efficacy as buprenorphine in the reduction of withdrawal symptoms (Cheskin et al., 1994; Ziaaddini et al., 2012). Clonidine treated patients however suffered from lower blood pressure compared to buprenorphine treated patients (Cheskin et al., 1994).

### Hematological, Biochemical and Histopathological Changes in Mitragynine and the Replacement Drugs

Biochemical and histopathological evaluations following mitragynine and/or the replacement drugs in blood and selected vital organs have also been conducted in the present study. In the hematological analysis (Table 2), MCHC increases in mitragynine-vehicle, mitragynine-0.1 mg/kg clonidine and mitragynine-1.0 mg/kg methadone. This might be due to the withdrawal effect that might be still exist in the body of the rats. Moreover, a substantial rise in MCHC level had also been reported in heroin and opium dependent and withdrawal groups (Haghpanah et al., 2010). In addition, the mitragynine-0.1 mg/kg clonidine treatment lowered platelet count level below the normal reference range without affecting other hematological parameters. This indicates that the rats might be affected with biological variations namely variability between individual rats and temporal variation (Peng et al., 2004), which warrants further details investigations.

In biochemical analysis (Table 3), mitragynine- 1.0 mg/kg methadone and mitragynine-0.8 mg/kg buprenorphine significantly reduced the total cholesterol level as compared to mitragynine-vehicle within the normal reference range value. A high level of total cholesterol has also been reported among kratom users who had a high daily mean frequency of kratom use (Leong Bin Abdullah et al., 2020).

Overall, a healthy set of organ cells except for lung can be observed in light microscopy examination of histopathology of all mitragynine replacement groups (Table 4). Previously, Macko et al. (1972) performed a subchronic study in rats and dogs in which no adverse effects were seen after oral administration of 5 or 50 mg/kg/day of mitragynine for six weeks, five days a week. Another subchronic study conducted by Sabetghadam et al. (2013) reported that mitragynine was relatively safe at lower subchronic doses (1–10 mg/kg, oral), but showed toxicity at the highest dose (subchronic 28 days: 100 mg/kg, oral) in the liver, kidney and brain. This dose caused also hematological and biochemical changes. All these toxicity data were reported after oral application though, which differs from the present study, which used i.p. applications. After i.p. administration, the primary route of absorption is through the mesenteric vessels, which drain into the portal vein and pass through the liver (Lukas et al., 1971). Substances administered i.p. can, therefore, undergo hepatic metabolism before reaching the systemic circulation (Turner et al., 2011). Moreover, in all treated groups, particularly the mitragynine-vehicle group, mild lung histopathological changes were observed compared with vehicle control group (Table 4), suggesting that the selected doses of drugs administration at determined duration, were too small to cause histopathological damage but sufficient to show signs of drug intoxication.

Opiates, stimulants, and cannabinoids are three classes of drugs that can cause respiratory manifestation (Glassroth et al., 1987). There are pulmonary patho-histological findings that have the direct effect on lung; edema, pulmonary hemorrhage and appear of siderophages, pulmonary artery medial hypertrophy, panacinar emphysema, bronchiolitis obliterans, interstitial pneumonia or fibrosis (Karch and Stephens, 2000). In addition, Awadalla and Salah-Eldin (2016) reported that opioids can cause a drop in plasma antioxidant levels, possibly indicating that the antioxidant defence mechanism against oxidative damage has failed. Indeed, oxidative stress during mitragynine withdrawal has been reported by Hassan et al. (2021). Therefore, the mild lung histopathological changes might be related to oxidative stress.

Opioid usage is inextricably connected to respiratory depression, or hypoventilation. However, it has an indirect effect on the lungs (Radke et al., 2014). Respiratory depression occurs when the body is unable to efficiently eliminate carbon dioxide. This can result in the lungs' inefficient utilisation of oxygen. As a result, the body produces more carbon dioxide and has insufficient oxygen. Basically, mu-opioid receptors are mostly found in the brainstem and are expressed on neurons that govern breathing (Boom et al., 2012). The activation of mu-opioid receptors causes opioid-induced analgesia as well as respiratory depression (Boom et al., 2012). Opioids exert their respiratory depressant effect via two distinct mechanisms: decreased chemoreceptor sensitivity and decreased activity in the central respiratory centres (White and Irvine, 1999). The carotid and aortic bodies, as well as the lungs, contain peripheral chemoreceptors. They boost signal transduction in response to decreased partial pressure of oxygen (pO_2_) or increased partial pressure of carbon dioxide (pCO_2_). The central receptors, which are located in the medulla but separate from the main respiratory centre, respond only to elevated pCO_2_ (Radke et al., 2014). Mu- and delta opioid receptors are found in the central respiratory regions of the medulla (Radke et al., 2014). The respiratory centre’s opioid activity induces a decrease in respiratory rate and tidal volume, both of which can contribute to a decrease in minute ventilation (White and Irvine, 1999). These effects are also dose-dependent where, at low dosages of opiates appear to decrease tidal volume, whereas larger doses appear to decrease both tidal volume and respiratory rate (Santiago and Edelman, 1985). In addition, in kratom itself, no single case can be solely attributed to respiratory failure, a sharp contrast to other opioids where respiratory depression is the most common cause of death (Kruegel and Grundmann, 2018). Plus, even though mitragynine activated the G-protein–mediated signaling pathway much like traditional opioids, it did not “recruit” β-arrestin-2 (Váradi et al., 2016), suggested that mitragynine has less side effect in terms of respiratory depression while it remains as a potent analgesic.

## Conclusion

In conclusion, four-day replacement therapy with available prescription drugs, methadone, buprenorphine, and clonidine, significantly attenuated mitragynine withdrawal signs in rats. This is the first study that reports these possible treatments for mitragynine withdrawal. As the present mitragynine substitution treatment was a preliminary study, further investigations would be required to confirm these findings in future.

## Data Availability

The raw data supporting the conclusions of this article will be made available by the authors, without undue reservation.
